# ERCC1 and BRCA1 mRNA expression levels in metastatic malignant effusions is associated with chemosensitivity to cisplatin and/or docetaxel

**DOI:** 10.1186/1471-2407-8-97

**Published:** 2008-04-11

**Authors:** Lifeng Wang, Jia Wei, Xiaoping Qian, Haitao Yin, Yang Zhao, Lixia Yu, Tingting Wang, Baorui Liu

**Affiliations:** 1 Department of Oncology, Drum Tower Hospital Affiliated to Medical School of Nanjing University & Clinical Cancer Institute of Nanjing University, Zhongshan Road 321, Nanjing 210008, China; 2 Department of Epidemiology and Biostatistics, Epidemiology and Biostatistics Graduate Program, Data Analysis Center, School of Public Health, Nanjing Medical University, Nanjing 210019, China

## Abstract

**Background:**

One of the major challenges in currently chemotherapeutic theme is lacking effective biomarkers for drug response and sensitivity. Our current study focus on two promising biomarkers, ERCC1 (excision repair cross-complementing group 1) and BRCA1 (breast cancer susceptibility gene 1). To investigate their potential role in serving as biomarkers for drug sensitivity in cancer patients with metastases, we statistically measure the mRNA expression level of ERCC1 and BRCA1 in tumor cells isolated from malignant effusions and correlate them with cisplatin and/or docetaxel chemosensitivity.

**Methods:**

Real-time quantitative PCR is used to analysis related genes expression in forty-six malignant effusions prospectively collected from non-small cell lung cancer (NSCLC), gastric and gynecology cancer patients. Viable tumor cells obtained from malignant effusions are tested for their sensitivity to cisplatin and docetaxel using ATP-TCA assay.

**Results:**

ERCC1 expression level is negatively correlated with the sensitivity to cisplatin in NSCLC patients (P = 0.001). In NSCLC and gastric group, BRCA1 expression level is negatively correlated with the sensitivity to cisplatin (NSCLC: P = 0.014; gastric: P = 0.002) while positively correlated with sensitivity to docetaxel (NSCLC: P = 0.008; gastric: P = 0.032). A significant interaction is found between ERCC1 and BRCA1 mRNA expressions on sensitivity to cisplatin (P = 0.010, n = 45).

**Conclusion:**

Our results demonstrate that ERCC1 and BRCA1 mRNA expression levels are correlated with *in vitro *chemosensitivity to cisplatin and/or docetaxel in malignant effusions of NSCLC and gastric cancer patients. And combination of ERCC1 and BRCA1 may have a better role on predicting the sensitivity to cisplatin than the single one is considered.

## Background

The lack of understanding and accounting for inter-patient variability in drug efficacy is one of the handicaps in the field of chemotherapy, which inevitably leads to the unpredictable disease responses and patient toxicities. New advanced technology, such as phamacogenomics, offers us a more efficient tool to explore the candidate genes that influence drug activity and toxicity. Such studies make it possible to perform tailor chemotherapy based on the specific genetic profile of individual patients [1,2]. Moreover, establishing any potent biomarkers for chemotherapy resistance will allow for the adoption of a better chemotherapeutic regimen, which will maximize efficacies and minimize toxicities of chemotherapy.

Genetic alterations are one of the major reasons for chemo-resistant in metastases[3]. Given the complicated biological mechanisms evolved in the disease progress and the heterogeneity between primary tumor and metastatic tumor[3,4], certain biomarkers, which have been proved to be effective to specific drug sensitivity in primary tumor-based studies, may not always serve the same way in metastases. For example, the higher expression of Xeroderma Pigmentosum A in metastatic effusion is associated with better response to chemotherapy in ovarian cancer patients, paradoxically to the results in primary tumor [5] However, low expression of thymidylate synthase (TS) in metastases is associated with response to 5-FU based therapy in advanced gastric disease [6,7], paralleling to those findings based on primary tumor tissues [8]. In order to develop better therapies and improve patients' outcome, one of the urgent work is to explore more effective biomarkers for chemosensitivity in metastases, which is the leading cause of cancer-related death in the world [4].

The excision repair cross-complementing group 1 gene (ERCC1), an essential member of the nucleotide excision repair (NER) pathway which accounts for the majority of platinum-DNA adduct repair, has been established as a useful molecular marker for NER activity. Early studies have shown that higher ERCC1 mRNA levels are associated with a more active DNA repair process in various tissues [9]. Interestingly, ERCC1 expression is also associated with cellular and clinical resistance to platinum compounds and to platinum-based chemotherapy, including lung and gastric malignancies [10-12]. Breast cancer susceptibility gene 1 (BRCA1), an essential component in multiple DNA damage repair pathways and pathways involved in cellular responses to microtubule damage, is considered to be a differential modulator of survival with cisplatin and taxanes [13-16]. Preclinical and clinical studies have reported that BRCA1 level is associated with cisplatin [14,17] and taxanes chemosensitivity [18,19]. Although above studies suggested that both ERCC1 and BRCA1 may serve as effective biomarkers for chemosensitivity in cancer patients with primary tumor, the information on these biomarkers is still limited in metastases.

Current study is therefore designed to explore the possibility of using ERCC1 and BRCA1 as two effective drug sensitivity biomarkers in malignant effusion specimens, in which all malignant cells are metastatic. We measured mRNA level of ERCC1 and BRCA1 in tumor cells isolated from malignant effusions and correlated them with cisplatin and/or docetaxel chemosensitivity *in vitro*. And we also verified the effect of combination ERCC1 and BRCA1 on the prediction of chemosensitivity to cisplatin.

## Methods

### Patients

All specimens and relevant clinical data were obtained from the department of oncology, respiratory and digestion, Drum Tower Hospital, Nanjing, China, during the period from June 2005 to December 2006. Malignant effusions were obtained from forty-six patients who have been histological or cytological diagnosis as stage IV malignant disease. Nine gastric cancer patients have received chemotherapy based on a semi-monthly regimen of 5-FU (5-fluorouracil), leucovorin and oxaliplatin before the appearance of effusions. Clinical characteristics of the patients are summarized in Table 1. In all specimens included in this study, cancer cells comprised at least 50% of the entire cells population based on cytology smears performed by the experienced pathologist. Informed consent was obtained from all patients and the protocols for this study were approved by the Human Research Protective Committee of our hospital.

**Table 1:** Demographic and clinical parameters of patients (n = 46)

	Patients	
Characteristics	No.	%
Age, years		
Median	63	
Range	38–87	
Sex		
Female	21	46
Male	25	54
Stage IV	46	100
ECOG performance status		
0–2	46	100
Primary Tumor		
NSCLC	20	43
Gastric	21	46
Gynecological	5	11
Sample type		
Pleural effusions	21	46
Peritoneal effusions	25	54
Pre-Chemotherapy		
Yes	9	20
No	37	80

Abbreviation: ECOG, Eastern Cooperative Oncology Group.

### Sample collection and processing

All patients received catheter placement using an indwelling 16 G single lumen central venous catheter (CVC) (Arrow™ 16 G CVC set Catheter, Arrow, USA). Effusions were collected in sterile drainage bag with heparin (10,000 U/L effusions) added previously, submitted to our laboratory within minutes and were processed immediately. Samples were centrifuged at 1000 g for 10 minute to collect cells. After Ficoll-Hypaque (specific gravity 1.077, Pharmacia) density centrifugation, the interface layer was collected. The quality and viability of the cell suspension were assessed by trypan blue dye exclusion and cytological examination. Cells were counted, washed and centrifuged for later ATP-TCA assay and RNA extraction.

### RNA isolation and cDNA synthesis

Total cell RNA was extracted using Trizol Reagent (Invitrogen, CA, USA) following the manufacture's protocol. cDNA was generated using Exscript™ RT reagent Kit (TaKaRa) according to manufacture's protocol.

### Real time PCR quantification

Quantitation of the genes of interest and an internal reference gene (β-actin) was done using a fluorescence based real-time detection method (Mx3000P Real Time PCR System, Stratagene). Briefly, 2 μl of cDNA were used for each RT reaction. The 20 μl PCR reaction mixture contained 1× primers & probe mixture (Applied Biosystems, Foster city, CA. Assay IDs: Hs00157415_m1 (ERCC1); Hs00173233_m1 (BRCA1); Hs99999903_m1(β-actin)), 1× Absolute QPCR Mix (ABgene, Surrey, UK). The PCR conditions were 50°C for 2 min, 95°C for 15 min, followed by 45 cycles at 95°C for 15 s and 60°C for 1 min. Each sample was assayed in triplicate with commercial RNA as positive and RNase-free water as negative control.

Relative gene expression quantifications were calculated according to the comparative Ct method using β-actin as an endogenous control and commercial RNA (Clontech) control as calibrators on each plate. Final results were determined by the formula 2^-ΔΔCt^[17] and were analyzed with the Stratagene analysis software.

### Evaluation of sensitivity to anti-cancer drugs using ATP-TCA

The chemosensitivity test was performed with primary tumor cells from malignant effusions as described before [20]. Briefly, assays were performed in 96-well polypropylene microplates(Corning-Costar 3790, NY, USA). Test drug concentrations were administrated in triplicate in six different concentrations: 0.234, 0.475, 0.95, 1.9, 3.8, and 7.6 μg·ml^-1 ^cisplatin (QiLu Pharma. Co., China) or 0.288, 0.575, 1.15, 2.3, 4.6, and 9.2 μg·ml^-1 ^docetaxel (Hengrui Pharma. Co., China). Subsequently, 100 μl of single cell suspension (2×10^5 ^/ml) in serum-free medium (CAM; DCS, Innovative Diagnostic Systeme, Hamberg, Germany) were added to each well. Two rows of the plate were reserved for controls, negative control (no drug, MO) and positive control (maximum ATP inhibitor, MI). After 6 days incubation at 37°C and 95% humidity in 5% CO_2 _atmosphere, intracellular ATP was extracted using the CellTiter-GIo Luminescent Kit (Promega, USA) according to the manufacturer's instructions and measured by microplate luminometer (Berthod Diagnostic system, Germany). A sensitivity index ranging from 0 to 600 for each drug was calculated by summing up the percentage of cell viability at the six drug concentrations tested [21]. Thus, a sensitivity index of 0 indicates maximal drug sensitivity, whereas a sensitivity index of 600 reflects minimal drug sensitivity.

#### Statistics

Spearman correlation coefficient was adopted to analysis the correlation between gene expression levels and drug sensitivity. ANOVA was used to evaluate the interaction of ERCC1 and BRCA1 on sensitivity to cisplatin. All values were based on two tailed statistical analysis. *P *value < 0.05 was considered statistically significant. Statistical analyses were done using SPSS 13.0 (SPSS Inc, Chicago, Illinois, USA).

## Results

### ATP-TCA

To determine the sensitivity to these drugs, primary tumor cells were isolated from malignant effusions and their chemosensitivity to cisplatin and docetaxel were assessed using the ATP-TCA assay. The serum free medium and polypropylene 96-well "U" microplates can enrich tumor cells up to 80–90%, reducing potential interference of non-tumor cells [20,22]. And we have also proved the raise of tumor cells number by the end of 6-days culture in our preliminary test. Due to the strict sterile operation, sample processing and only the specimens containing at least 50% cancer cells enrolling, assays were successfully performed on all samples. Table 2 summarized the result of ATP-TCA on different subsets.

**Table 2:** In vitro chemosensitivity assay results

Cytotoxic drug	Total	NSCLC	Gastric	Gynecological
	
	Sensitivity Index of Median (range), No. patients			
Docetaxel	455 (206–600), 46	422 (206–566), 20	513 (382–600), 21	364 (206–438), 5
Cisplatin	442 (184–600), 46	445 (184–558), 20	455 (273–600), 21	387 (301–464), 5

### ERCC1 expression and cisplatin chemosensitivity

**Figure 1:**
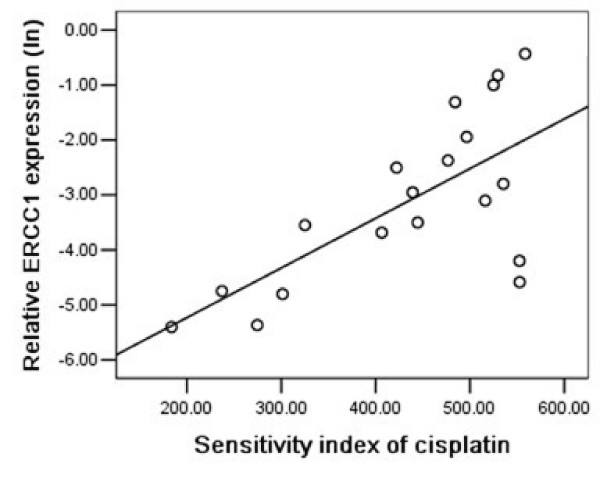
**A sensitivity index of 0 indicates maximal drug sensitivity, whereas a sensitivity index of 600 reflects minimal drug sensitivity.** Relative ERCC1 mRNA expression (logarithmically transformed) was positively correlated with resistance to cisplatin in 19 NSCLC patients (P = 0.001, r = 0.685).

**Figure 2:**
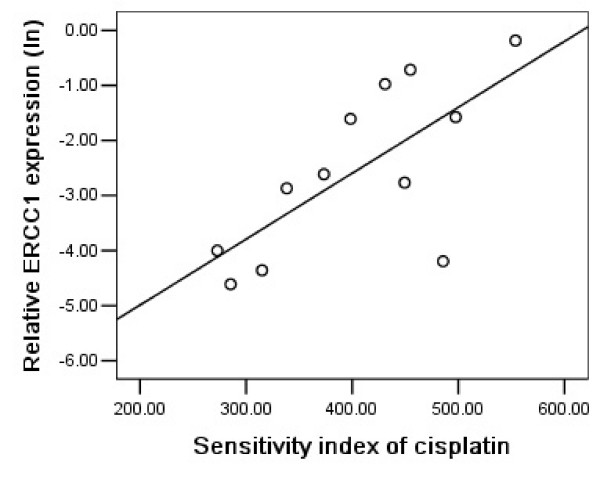
ERCC1 mRNA expression levels were significant association with sensitivity to cisplatin in 12 untreated gastric cancer patients (P = 0.014, r = 0.685).

Except one specimen from NSCLC patients, ERCC1 were amplified in all other samples. The median ERCC1 mRNA expression was 0.057 (range: 0.005–0.831, n = 45). Figure 1 shows a significant correlation between ERCC1 mRNA expression and sensitivity to cisplatin in malignant pleural effusions of NSCLC patients (P = 0.001, r = 0.685). Patients with lower ERCC1 mRNA expression levels had a higher sensitivity to cisplatin compared with those with higher expression levels. However in gastric group, we did not find a significant association between ERCC1 mRNA expression and cisplatin chemosensitivity (P = 0.482, r = 0.162). Considering ERCC1 expression is up-regulated in response to cisplatin exposure [23] and 9 patients have been treated with oxaliplatin before, we deduced that previous exposure to platinum might induce alterations on ERCC1 gene expression, which could be different according to individual patient. When nine pre-treated patients were excluded from this group, a significantly correlation between the level of ERCC1 and chemosensitivity to cisplatin was found in untreated gastric patients (P = 0.014, r = 0.685; Figure 2).

### BRCA1 expression and cisplatin chemosensitivity

**Figure 3:**
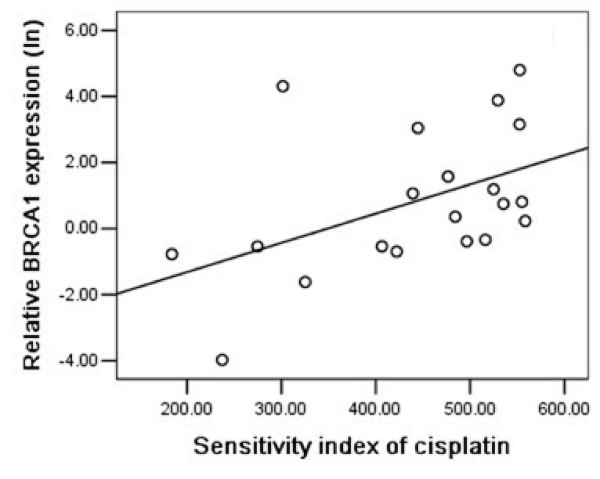
Relative BRCA1 mRNA expression levels (logarithmically transformed) were positively correlated with resistance to cisplatin in 20 NSCLC patients (P = 0.014, r = 0.541).

**Figure 4:**
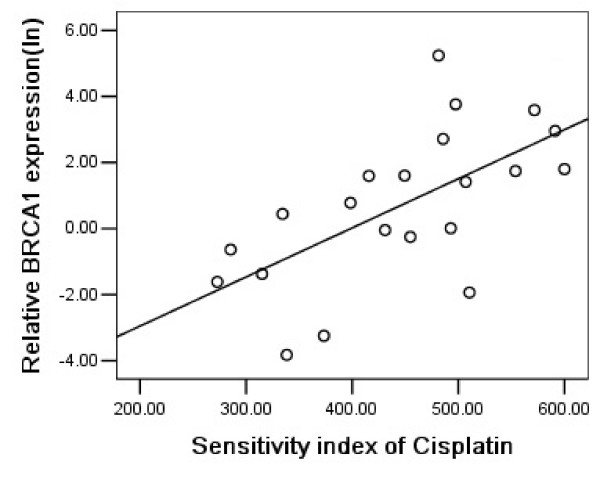
Relationship between BRCA1 and sensitivity to cisplatin in 21 gastric cancer patients (P = 0.002, r = 0.624).

We next analyzed the correlation between BRCA1 mRNA expression and cisplatin chemosensitivity in malignant effusions. BRCA1 mRNA expression was detectable by quantitative RT-PCR in all samples. The median level was 2.209×10^-3 ^(range, 0.019–188.645×10^-3^, n = 46). As shown in Figure 3 and 4, BRCA1 mRNA expression levels were inversely correlated with sensitivity to cisplatin in malignant pleural effusions of NSCLC patients (P = 0.014, r = 0.541; Figure 3) and in ascites of gastric patients (P = 0.002, r = 0.624; Figure 4).

### BRCA1 expression and docetaxel chemosensitivity

**Figure 5:**
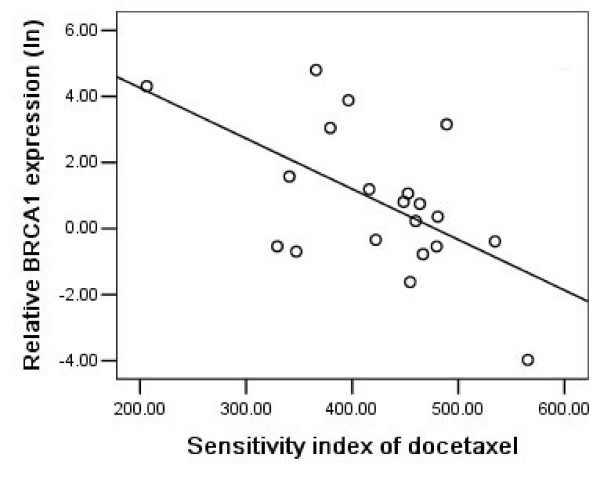
BRCA1 mRNA expression levels (logarithmically transformed) were negatively correlated with resistance to docetaxel in 20 NSCLC patients (P = 0.008, r = -0.573).

**Figure 6:**
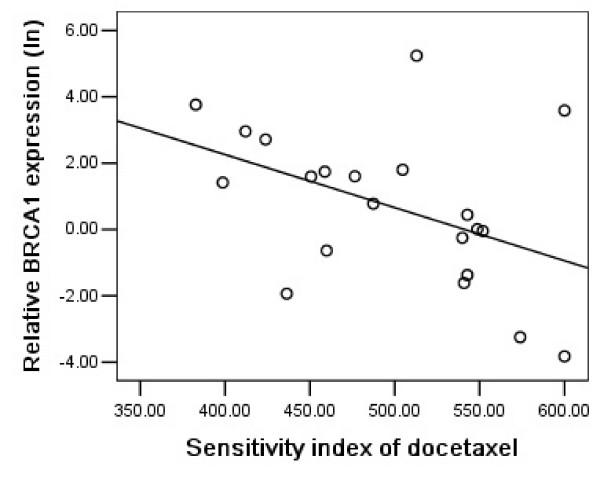
Relationship between BRCA1 mRNA expression and sensitivity to docetaxel in 21 gastric cancer patients (P = 0.032, r = -0.468).

On the opposite site of its prediction value for cisplatin sensitivity, BRCA1 mRNA expression levels in malignant effusions were positively correlated with sensitivity to docetaxel, in which patients with higher BRCA1 mRNA expression levels had a higher sensitivity to docetaxel compared with those with lower expression levels (NSCLC: P = 0.008, r = -0.573, Figure 5; gastric: P = 0.032, r = -0.468; Figure 6). We further noticed that the correlation between BRCA1 mRNA expression and docetaxel chemosensitivity in gastric group was due to the two extreme points, and that a higher number of samples would be necessary to confirm it.

### Combination of two genes and sensitivity to cisplatin

As both ERCC1 and BRCA1 were correlated with cisplatin chemosensitivity, we further investigated the effect of combination of them on the sensitivity to cisplatin. A significant interaction was found between BRCA1 and ERCC1 gene expression on the sensitivity to cisplatin in total group (P = 0.010, n = 45) and gastric group (P = 0.027, n = 21). When both ERCC1 and BRCA1 were lowly expressed, the sensitivity to cisplatin was improved and which was higher than the sum of the degrees when only single marker was lowly expressed. This could be explained as combination of low expression levels of ERCC1 and BRCA1 correlated with more sensitivity to cisplatin than single marker being considered. Similar trend was also found in NSCLC group without statistical significance (P = 0.12, n = 19).

## Discussion

The primary purpose of this study was to determine the correlation between ERCC1 and BRCA1 mRNA expression and sensitivity to cisplatin and/or docetaxel in special metastases, malignant effusions. We use body cavity as a specific metastatic site in current study based on following reasons. Firstly, tumor cells in malignant effusions have distinct characteristics such as loss of interaction with stromal and endothelial cells, reduced availability of oxygen and nutrients [24]. Secondly, it is easy, non-invasive and repeatable to obtain effusion samples from inoperable lung or gastric cancer patients with malignant effusions [25]. Finally, malignant effusion is a complication of various advanced malignant disease. Approximately half of all patients with metastatic cancer develop a malignant effusion at the course of disease [26].

ATP-TCA, a well established *in vitro *assay, has been demonstrated as a valuable tool for the evaluation of chemosensitivity in solid tumors, as well as been shown to allow a prediction of clinical results [21,27]. In the present study, ATP-TCA was performed to evaluate the single agent chemosensitivity in a consecutive series of 46 malignant effusions. By using the in vitro assay, we excluded the interference from other systematic therapy such as combination of other drugs and potential prognostic effects in vivo, thus making the assessment between particular gene expression and a given drug more precise. However, chemosensitivity results from an *in vitro *study can not be directly translated into clinical practice because the chemoresponse does not depend on intrinsic chemosensitivity alone. Our future study will focus on the confirmation of the relationship between these two genes expression in malignant effusions and patients' clinical response in clinical trial.

We observed a clear correlation between ERCC1 and BRCA1 mRNA expression level and chemosensitivity to cisplatin, one of the most widely used agent in both NSCLC and gastric cancer. Both ERCC1 and BRCA1 were negatively correlated with cisplatin sensitivity in pleural effusions of NSCLC patient. In addition, BRCA1 mRNA expression was negatively correlated with cisplatin sensitivity in peritoneal effusions of gastric cancer patients, while ERCC1 was correlated with cisplatin sensitivity only in specimens of untreated gastric patients. Unlike ERCC1, BRCA1 is involved in both homologous recombination and nonhomologous end joining of double-stranded DNA breaks and also in NER of DNA adducts [15,16]. It is reasonable to speculate that the different mechanisms of these two genes on DNA repair might be a possible explanation for this disparity.

In comparison to the evidence linking ERCC1 to cisplatin sensitivity, the link between BRCA1 and response to taxane remains to be documented. We demonstrated a positively correlation between BRCA1 mRNA expression and docetaxel sensitivity in malignant effusions in both NSCLC and gastric patients. Our study showed that patients with higher BRCA1 level in malignant effusions are more sensitive to docetaxel than those with lower level. This finding is in line with the previous reports [18,19]. Knockdown of BRCA1 gene induce a 50-fold increase in resistance to paclitaxel agents [18]. A recently study show that absence of BRCA1 expression is a predictor of shorter TTP (time to progression) in advanced breast cancer patients treated with taxane-based therapy [19]. Collectively, our findings supported the hypothesis that BRCA1 and ERCC1 mRNA expression levels were correlated with sensitivity to docetaxel and/or cisplatin in metastatic effusions, which is in consistent with previous findings based on primary tumor.

Our second aim was to study whether the combination of ERCC1 and BRCA1 could do better on predicting the chemosensitivity to cisplatin. We observed the significant interaction of BRCA1 and ERCC1 mRNA expression levels on sensitivity to cisplatin in total and gastric cancer patients. However, we did not obtain the same conclusion in lung cancer patients. This might due to the small sample size and over-dispersion data, which would restrict the statistical power to identify interaction effect in that panel. According to our results, the combination of two biomarkers, ERCC1 and BRCA1, may play a better role in predicting the cisplatin chemosensitivity than the single one being considered.

## Conclusion

In summary, our data demonstrated for the first time that ERCC1 and BRCA1 mRNA expression levels were correlated with cisplatin and/or docetaxel in the *ex vivo *study of malignant effusions. Combination of ERCC1 and BRCA1 mRNA expression may have an interaction prediction on the sensitivity to cisplatin.

## Competing interests

The author(s) declare that they have no competing interests.

## Authors' contributions

LFW preformed the ATP-TCA assay, real-time PCR, and wrote the paper. JW carried out the RNA extraction. XPQ participated in drafting the manuscript. HTY collected clinical samples and relevant clinical data. YZ carried out the statistical relevant work and study design. LXY performed the tumor cell separation from effusions. TTW participated in the specimen collection and transportation. BRL designed the study and instructed the research work. All authors read and approved the final manuscript.

## Pre-publication history

The pre-publication history for this paper can be accessed here:


